# Subcellular Distribution of Mitochondrial Ribosomal RNA in the Mouse Oocyte and Zygote

**DOI:** 10.1371/journal.pone.0001241

**Published:** 2007-11-28

**Authors:** Youichirou Ninomiya, Shizuko Ichinose

**Affiliations:** 1 Mammalian Development Laboratory, Department of Zoology, University of Oxford, Oxford, United Kingdom; 2 Instrumental Analysis Research Centre, Tokyo Medical and Dental University, Bunkyo, Tokyo, Japan; Max Planck Institute of Molecular Cell Biology and Genetics, Germany

## Abstract

Mitochondrial ribosomal RNAs (mtrRNAs) have been reported to translocate extra-mitochondrially and localize to the germ cell determinant of oocytes and zygotes in some metazoa except mammals. To address whether the mtrRNAs also localize in the mammals, expression and distribution of mitochondrion-encoded RNAs in the mouse oocytes and zygotes was examined by whole-mount *in situ* hybridization (ISH). Both 12S and 16S rRNAs were predominantly distributed in the animal hemisphere of the mature oocyte. This distribution pattern was rearranged toward the second polar body in zygotes after fertilization. The amount of mtrRNAs decreased around first cleavage, remained low during second cleavage and increased after third cleavage. Staining intensity of the 12S rRNA was weaker than that of the 16S rRNA throughout the examined stages. Similar distribution dynamics of the 16S rRNA was observed in strontium-activated haploid parthenotes, suggesting the distribution rearrangement does not require a component from sperm. The distribution of 16S rRNAs did not coincide with that of mitochondrion-specific heat shock protein 70, suggesting that the mtrRNA is translocated from mitochondria. The ISH-scanning electron microscopy confirms the extra-mitochondrial mtrRNA in the mouse oocyte. Chloramphenicol (CP) treatment of late pronuclear stage zygotes perturbed first cleavage as judged by the greater than normal disparity in size of blastomeres of 2-cell conceptuses. Two-third of the CP-treated zygotes arrested at either 2-cell or 3-cell stage even after the CP was washed out. These findings indicate that the extra-mitochondrial mtrRNAs are localized in the mouse oocyte and implicated in correct cytoplasmic segregation into blastomeres through cleavages of the zygote.

## Introduction

Producing different cell types through segregation of specific cellular components is a key aspect of embryogenesis, which is implemented at the very beginning of the embryogenesis in some species. The anterior-posterior axis is laid down within *Nematoda* zygote immediately after sperm entry following which the zygote goes through a series of asymmetric cleavage to produce certain progenitor cells including germ cells [1]. In *Drosophila*, several gene products are regionally localized in the oocyte cytoplasm to define the body plans of the future embryo [2], [3] and specify the complement of future germ cells [4]. These are regarded as pre-patterned oocytes and zygotes since different domains of cytoplasm harbour distinct developmental properties. Even in vertebrates such as *Xenopus*, several RNAs are reported to subcellularly localize in the oocyte [5]–[10].

Localization of germ plasm and its subsequent segregation to germ cells in early embryogenesis has been investigated particularly thoroughly. In both *Drosophila* and *Xenopus*, germ plasm is localized in the oocyte, where it is clearly defined as a region rich in mitochondria and possessing “nuage” or germinal bodies, polar granules in *Drosophila* and germinal granules in *Xenopus*, which characterize prospective germ cells in developing embryos [4], [9]. The germinal body contains nucleic acids and that germ cell determinant capability is lost when the germ plasm is irradiated with short-wavelength UV, but can be restored if intact germ plasm is transplanted into the irradiated zygotes [11]. Surprisingly the nucleic acid in question is mitochondrial ribosomal RNA (mtrRNA) that lies outside this organelle [12], [13]. MtrRNA injected into the UV-irradiated *Drosophila* zygote restored the formation of pole cells [14] indicating that this class of RNA may be essential for pre-patterning of the germ plasm in *Drosophila*, sea urchin [15] and *Xenopus* [16] zygote.

Although some genes products are localized in mammalian oocytes [17], [18], it is widely accepted that early development is essentially regulative rather than pre-patterned in mammals [19]–[21]. While mechanical perturbation to the cytoplasm, for instance, does not perturb embryogenesis [22]–[24], there are a growing number of findings that challenge the absence of pre-patterning in mammals [25]. Moreover, the existence of a regulative capacity does not necessarily rule out pre-patterning of the oocytes [26]–[29]. Several mammalian homologues to the posterior group genes of *Drosophila* have been identified, and many of them are expressed in the mouse primordial germ cells [30]. Little is, however, known about the molecular organization of the mouse oocyte and zygote in which no distinctive germ plasm has been identified and the occurrence of extra-mitochondrial mtrRNAs has not been reported. The purpose of the present study was therefore to investigate if such mtrRNA is localized in the mouse oocyte or zygote and, if so, whether has any role in patterning the embryo. The study revealed that the mtrRNAs are localized extra-mitochondrially in the mouse oocyte and implicated in correct cytoplasmic segregation into blastomeres through cleavages of the zygote.

## Materials and Methods

### Collecting and culturing mouse oocytes and zygotes

All oocytes and zygotes were obtained from PO (Pathology, Oxford) albino closed-bred mice. Media and conditions for recovery and culture of oocytes and zygotes, and for strontium-activation for haploid parthenogenetic development, were as reported previously [27], [31]. All experiments regarding animals were carried out under the UK legislation for animal welfare (Animals (Scientific Procedures) Act 1986, Home Office, UK, scienceandresearch.homeoffice.gov.uk/animal-research/).

Chloramphenicol (CP, SIGMA, www.sigmaaldrich.com), cycloheximide (CH, Merck, www.merck4biosciences.com) and á-amanitin (AM, Merck) were dissolved into dimethylsulfoxide (DMSO, SIGMA) to make stock solutions. Stock solutions were diluted 1∶100 in KSOM-AA medium to obtain the following final concentrations in the medium; CP 200 µg/mL [32], CH 10 µg/mL [33] and AM 100 µg/mL [34]. These final concentrations were determined by referring to previous reports. For vehicle control, 1% DMSO in KSOM-AA was used.

MitoTracker Red H_2_-CMXRos (Molecular Probes, probes.invitrogen.com), which is chemically reduced and non-fluorescent unless oxidized [35], was prepared according to the manufacturer's instruction and applied final concentration of 400 nM to the treated conceptuses for 30 minutes at 37°C before fixation. The conceptuses were fixed in 4% paraformaldehyde (PFA) in calcium- and magnesium-free Dulbecco's phosphate buffered saline (PBS(-)) for 5 minutes at room temperature, then washed with PBS(-) and mounted on glass slides with 60% glycerol in PBS(-). Fluorescent images were photographed using a CCD camera (MagnaFire, Optronics, www.optronics.com) on a conventional epifluorescent microscope (Axioplan, Zeiss, www.zeiss.com).

### Morphometric analysis of 2-cell conceptuses

Cultured conceptuses were treated with acidified-Tyrode's saline to remove the zonae pellucidae then further incubated in calcium-free OC medium supplemented with EGTA for 30 minutes at 37°C to release membrane tension and leave blastomere adherence [36]. After treatment, conceptuses were incubated in KSOM-AA medium for 30 minutes at 37°C before being fixed and processed as described in previous section. The specimens were photographed using a digital camera (Coolpix 995, Nikon, www.nikon.com) on a differential interference contrast microscope (DMLB, Leica, www.leica-microsystems.com), and the photomicrographs were analyzed with the ImageJ software [37], [38] runs on a Macintosh personal computer (PowerMac G4, Apple, www.apple.com).

### Whole-mount in situ hybridization

Mitochondrial DNA was isolated from E6.5-7.5 (plug day noon = E0.5) mouse conceptuses by alkaline hydrolysis protocol designed for purification of plasmids from *Escherichia coli* (QIAprep Spin, QIAGEN, www1.qiagen.com). Using the purified DNA as a template, the following PCR primers containing partial T3 and T7 promoter (below, small caps) were used to generate templates for antisense and sense RNA probes.

1. 837 bp fragment of 12S rRNA fwd-pt-T3 cactaaagggAGGTTTGGTCCTGGCCTTAT, rev-pt-T7 cactatagggCGGTGTGTGCGTACTTCATT
2. 883 bp fragment of 16S rRNA fwd-pt-T3 cactaaaggGTACCGCAAGGGAAAGATGA, rev-pt-T7 cactatagggCAGTTGGACCCTCGTTTAGC
3. 656 bp fragment of ATPase 6 (ATP6) fwd-pt-T7 cactatagggcGAACGAAAATCTATTTGCCTCA, rev-pt-T3 cactaaaGGGCTTACTAGGAGGGTGAATACG
4. 869 bp fragment of Cytochrome *c* oxidase subunit 1 (Cox1) fwd-pt-T7 cactatagggcGGTCAACCAGGTGCACTTTT, rev-pt-T3 cactaaagggTTACCTCCGTGTAGGGTTGC

These DNA fragments were further amplified using the following adaptor primers to install full T3 and T7 promoter sequences into the fragments, AATTAACCCTCACTAAAGG for T3 and GTAATACGACTCACTATAGGGC for T7. All four PCR-generated templates were subjected to sequencing (BigDye Terminator v1.1 Cycle Sequencing Kit, Applied Biosystems, www.appliedbiosystems.com) and homology search (Nucleotide-nucleotide Basic Local Alignment Search Tool, National Center for Biotechnology Information, www.ncbi.nlm.nih.gov) to confirm the relevant gene identities and specificities. The 2.4 kbp PCR-generated cDNA of mouse EMK (ELKL Motif Kinase) cloned into pBluescript KS+ (mEMK c24, a gift from Prof. Ohno) was also used. The expression of 12S and 16S rRNAs, ATP6, Cox1 and mEMK mRNAs and Cytochrome *b* mRNA in the mouse MII oocytes had been confirmed by either blot hybridization [39] or EST analysis [40]. For digoxigenin (DIG) labeled *in vitro* transcription, T3 and T7 RNA polymerases (Roche, www.roche-applied-science.com) were used according to the manufacturer's instruction. Whole-mount *in situ* hybridization (ISH) for oocytes and zygotes was done as described in previous report [41].

For scanning electron microscopic (SEM) visualization of the ISH, frozen sections were prepared in accordance with previous report [42] with some modifications. The whole-mount hybridized and washed oocytes were embedded into 2% low-melting point temperature agarose (NuSieve GTG, FMC, www.fmc.com) in PBS(-), immersed in 1.8 M sucrose containing 20% polyvinylpyrrolidone in PBS(-) for 24 hours and quickly frozen in liquid nitrogen. Frozen sections with a thickness of less than 1 µm were cut by an ultramicrotome (Ultracut S, Reichert, www.leica-microsystems.com) equipped with a low-temperature sectioning system (FCS, Reichert). Gold colloidal particles (15 nm in diameter) conjugated anti-DIG antibody (Aurion, www.aurion.nl) was applied to the sections, which were then fixed in 2.5% glutaraldehyde in 0.2 M phosphate buffer (pH 7.4, PB), post-fixed in 1% OsO_4_ in PB, dehydrated in a graded series of ethanol and dried by a critical point drying apparatus (HCP-2, Hitachi, www.hitachi-hitec.com) with liquid CO_2_. The specimens were coated with thin osmium layer using an osmium plasma coater (OPC80N, Filgen, www.filgen.jp) and examined under a field emission type scanning electron microscope (S-4500, Hitachi) with a YAG backscatter electron detector [43]. The backscattered images were gray scale inverted and superimposed with the SEM images obtained from same field using a photo editing software (Photoshop CS, Adobe, www.adobe.com).

### Whole-mount immunofluorescence

Oocytes and zygotes were fixed in 4% PFA on ice for at least overnight, then washed in PBS(-) containing 0.1% Tween 20 (PBT). Washed specimens were transferred to 0.2 ml PCR tubes containing 100 µl of 10 mM citrate buffer (8.2 mM tri-sodium citrate and 1.8 mM citrate), and antigen-retrieved at 97°C for 30 minutes using a PCR machine. Prior to applying primary antibodies, specimens were blocked for at least 60 minutes on ice with 10% heat-inactivated FCS and 2 mg/mL of bovine serum albumin (BSA fraction V, SIGMA) supplemented PBT. Anti-mitochondrion specific heat shock protein 70 (mtHSP70) antibody (clone JG1, Abcam, www.abcam.com) and anti-á-tubulin (clone YOL 1/34, Abcam) antibody were diluted 1:250 in the blocking solution and applied to the specimens on ice overnight. Immunolocalizations were visualized via Alexa Fluor 546 conjugated anti-mouse IgG F(ab')2 (for mtHSP70, Molecular Probes) and Alexa Fluor 488 conjugated anti-rat IgG (for á-tubulin, Molecular Probes) diluted 1∶500 in PBT and applied for 2 hours at room temperature. Nuclei were counterstained with 20 µM Hoechst 33342 (Calbiochem, www.calbiochem.com) in PBT. The specimens were mounted on glass slides with 60% glycerol in PBS(-) then subjected to a laser confocal scanning microscopy (LSM510Meta, Zeiss) for optical sectioning at 1 µm thickness.

## Results

### Distribution of mitochondrial 16S rRNA

**Figure 1:**
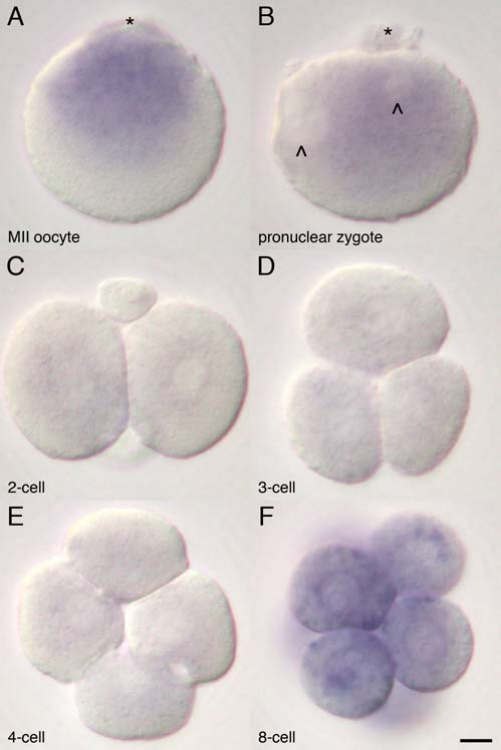
Subcellular distribution of 16S rRNA in the mouse oocyte and zygotes. Higher magnification of the MII oocyte (A) shows a predominant distribution of the 16S rRNA in animal hemisphere (asterisk: position of MII spindle). Distribution of the16S rRNA is rearranged to peri-pronuclei (chevrons) accumulation toward the polar body (asterisk) on the zygote (B), and the intensity decreases around the first cleavage (C). The amount of 16S rRNA remains low during the second cleavage (D and E) and increases after the third cleavage (F). Bar = 10 µm

From second meiotic metaphase arrested (MII) oocyte through to 8-cell morula stage conceptuses, both 12S and 16S rRNAs were localized identically but the ISH staining intensity of 12S rRNA (Figure S1A) was slightly weaker than that of 16S rRNA (Figure S1B). Considering single pre-processed mitochondrial transcripts are split into both mtrRNAs, this might be due to a difference of hybridization efficiencies between two probes rather than a difference of the actual amount of mtrRNAs. Therefore only ISH results of 16S rRNA are represented further. In the MII oocyte, the 16S rRNA was distributed at the presumptive animal hemisphere region-the half of the oocyte toward the MII spindle (Figure 1A, asterisk and S2B), the vegetal hemisphere being almost devoid of such RNA (Figure 1A and S2B). After fertilization, the distribution of the 16S rRNA changed to being localized around the pronuclei of the zygote (Figure 1B). Closer examination revealed that it lay preferentially between the pronuclei (Figure 1B, chevrons) and second polar body (Figure 1B, asterisk). The intensity of staining decreased after the first cleavage (Figure 1C) and remained low until the 4-cell stage (Figure 1D and 1E), before gradually increasing after the third cleavage (Figure 1F). Expression of ATP6 (Figure S1C) and Cox1 (Figure S1D) were undetectable during these stages.

To test the efficiency of this ISH methodology, localization of transcripts of mEMK, a mouse homologue of *Nematoda par-1* [44], [45], was also examined on the oocytes and zygotes (Figure S1E). As shown in the figure, the mEMK transcripts were distributed ubiquitously in the MII oocyte and zygote and dramatically decreased in abundance after the first cleavage. This pattern of expression of mEMK during these stages accords well with previous report in which RT-PCR was used to detect its transcripts [46]. This suggests that the negative results with ATP6 and Cox1 are due to difficulty of probe penetration rather than limited sensitivity of the ISH.

**Figure 2:**
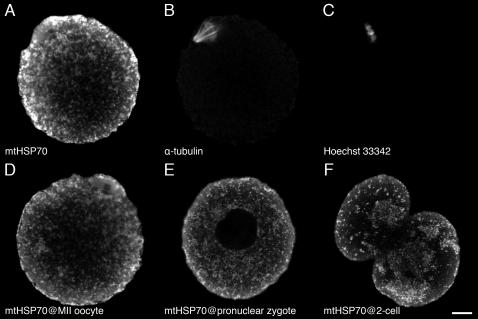
Subcellular distribution of mitochondrion-specific heat shock protein 70 in the mouse oocytes and zygotes. MtHSP70 is evenly distributed throughout the cytoplasm (A and D). Anti-á-tubulin (B) and Hoechst 33342 (C) fluorescence indicate an orientation of the oocyte as simultaneously visualized with anti-mtHSP70 immunofluorescence (A). Anti-mtHSP70 immunofluorescence is still evenly distributed the cytoplasm of zygote, except pronucleus where immunofluorescence is devoid (E). The immunofluorescence begins to aggregate in some places of the cytoplasm after the first cleavage (F). Bar = 10 µm

If the ISH for 16S rRNA is able to detect such RNAs within mitochondrion, their distribution should coincide with that of mitochondria. Immunofluorescent staining against mtHSP70 [47] clearly demonstrates that it is not the case. Consistent with previous reports where a mitochondrion-specific fluorescent dye was used [48], [49], the immunofluorescence showed mtHSP70 to be distributed not only around MII spindle (Figure 2B and 2C) but evenly throughout the cytoplasm of MII oocytes (Figure 2A and 2D) and zygote (Figure 2E). At the 2-cell stage when the ISH staining intensity of the 16S rRNA had decreased, the immunofluorescence was firmly visible in the cytoplasm (Figure 2F). Hence it is likely that the DIG-labeled probes could have difficulties to penetrate into the mitochondria under the ISH conditions used in this study.

**Figure 3:**
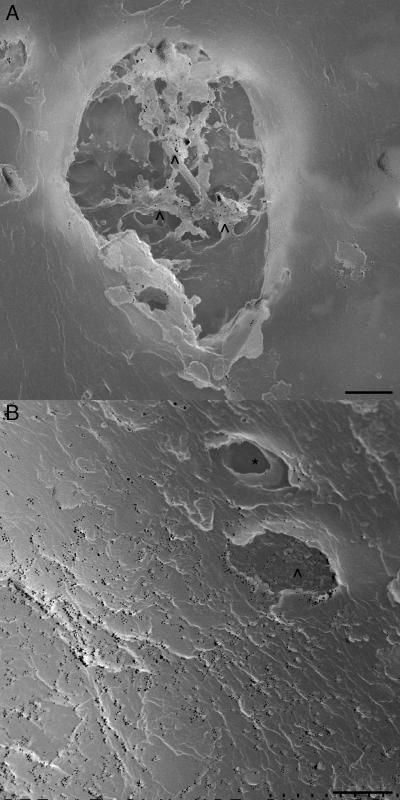
Localization of 16S rRNA at a sub-endoplasmic organelle level. The electron microscopic visualization of the ISH reveals the localization of 16S rRNA in MII oocyte at a sub-endoplasmic organelle level. Since the specimens are sectioned, only exposed interior of the organelle can be seen as concave structures in a SEM image. Gold colloidal particles (15 nm, small black dots) are visualized via a backscatter electron detector and seen to accumulate on the cristae (chevrons) of a mitochondrion (A). Surrounding cytoplasm is almost deserted by such particles. In certain area of the MII oocyte, the gold colloidal particles, however, spread extra-mitochondrially (B). The cytoplasm is more abundant in the particles than the interior of mitochondrion (chevron). In contrast to a structured interior surface of the mitochondrion, the endoplasmic reticulum has a smooth surface of interior (asterisk), which is devoid of the particle. Bars = 0.5 µm

The combination of ISH and SEM further provided strong evidence showing few DIG-labeled probes could penetrate into the mitochondria. A small number of gold colloidal particles are observed on tubular cristae [50], [51] of the mitochondrion (Figure 3A, chevrons), whereas the surrounding cytoplasm is almost deserted by the particles. The ISH-SEM also revealed that the 16S rRNAs are extra-mitochondrially distributing at the certain place of cytoplasm (Figure 3B and S2D). In these SEM fields, the particles are wide spread including inside the mitochondrion (Figure 3B, chevron), except the endoplasmic reticulum (Figure 3B, asterisk) in which the 16S rRNA is not expected to present.

### Distribution dynamics of the 16S rRNA after parthenogenetic oocyte activation

**Figure 4:**
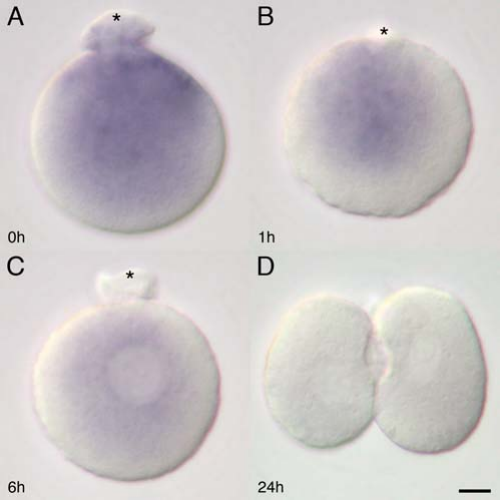
Subcellular distribution of 16S rRNA in the mouse parthenotes. The 16S rRNA ISH on strontium-activated *in vitro* parthenotes show similar distribution pattern to the *in vivo* zygotes. Strong distribution is visible around the second polar body (asterisk) extruding site of the oocyte immediately after (0 hour) the activation (A). On completion of the polar body extrusion, the distribution of 16S rRNA is rearranged to peri-pronuclei accumulation toward the polar body (asterisk) on the parthenote (B). The ISH staining intensity has already begun to decrease 6 hours after the activation (C). The staining intensity is weak (D) after the first cleavage. Bar = 10 µm

*In vitro* activation of oocyte revealed that the rearrangement in distribution of 16S rRNA in zygotes occurs independently of the presence of sperm components. Thus, the strontium-activated haploid parthenotes showed a similar distribution pattern and dynamics of the 16S rRNA (Figure 4) to that of the zygotes obtained by fertilization *in vivo* (Figure 1B and 1C). While the second polar body extrusion was taking place, the distribution of 16S rRNA remained predominantly in the animal hemisphere (Figure 4A). This predominant animal hemisphere distribution was succeeded by a peri-pronuclear one on completion of the polar body extrusion (Figure 4B). The amount of 16S rRNA in parthenotes began to decrease by 6 hours after activation (Figure 4C), and the staining intensity was faint after first cleavage (Figure 4D).

### Effect of inhibitor for mitochondrial translation

Chloramphenicol (CP) specifically inhibits prokaryotic translation and therefore serves as a specific inhibitor of mitochondrial translation in eukaryotes. Along with CP, cycloheximide (CH), a general inhibitor for translation, and á-amanitin (AM), a transcription inhibitor, were also applied to the late pronuclei stage zygote (around E0.75). In line with several other studies reporting that zygotic gene expression generally begins at 2-cell stage in mice [52]–[54], AM did not prevent the first cleavage, whereas CH prevented the first cleavage with the zygote in an elongated or ellipsoidal shape presumably arrested in the beginning of mitosis (Table 1). This indicates that *de novo* protein synthesis from maternally deposited mRNAs is required for completing the first cleavage.

**Table 1:** Effects of various inhibitors on the first cleavage of mouse zygotes

stage	1-cell	2-cell	dead	total
AM	1	44	1	46
vehicle	2	29	0	31
CH	48	25	12	85
vehicle	16	66	2	84
CP	14	135	1	150
vehicle	6	128	0	134

Late pronuclei stage zygotes (around E0.75) were cultured for 24 hours in presence of following inhibitors; AM: á-amanitin 100 µg/mL, CH: cycloheximide 10 µg/mL, CP: chloramphenicol 200 µg/mL, vehicle: 1% DMSO as vehicle control. Numbers of conceptuses reaching denoted cleavage stages were counted and presented. Numbers of independent trials: 5 for AM, 8 for CH and 14 for CP.

**Figure 5:**
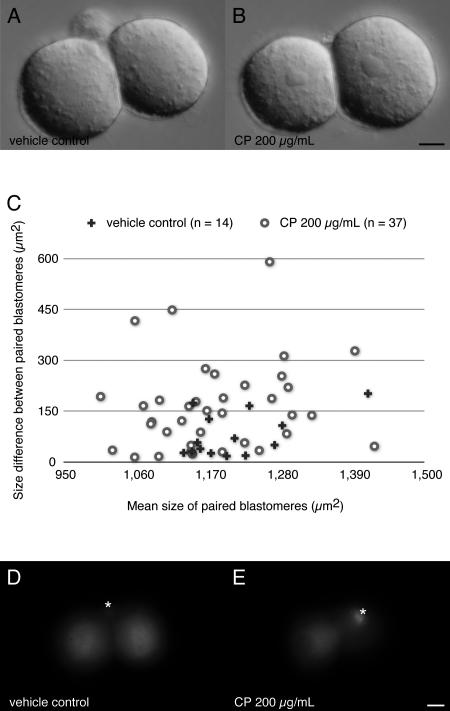
Effect of inhibitor for mitochondrial translation on the late pronuclei stage zygotes. Blastomeres of the conceptuses at 2-cell stage after 24 hours *in vitro* culture in presence of either 1% of DMSO vehicle control (A) or 200 µg/mL of CP (B) show a size difference in these 2 treatments. Outlines of each blastomere are traced on the microphotographs then converted to the areas using ImageJ software. Pair values of the area are processed to calculate “Mean size of paired blastomeres” and “Size difference between paired blastomeres” and plotted on a scatter diagram (C). Mann-Whitney's *U* tests indicate there is no significant difference in “Mean size” between the 2 treatments (*U* = 206, level of significance 0.263), however statistical difference in “Size difference” between them (*U* = 150, level of significance 0.021). For the raw data set see Table S1. MitoTracker Red H_2_-CMXRos staining at 2-cell stage after 24 hours treatment with either 1% of DMSO vehicle control (D) or 200 µg/mL of CP (E) indicate no visual sign of retardation of the mitochondrial oxidization capability. Polar bodies (asterisks) also indicate retaining active mitochondria. Bars = 10 µm

While exposure of late zygote to CP did not prevent first cleavage (Table 1), it resulted in many 2-cell conceptuses showing unequal sized blastomeres (Figure 5A and 5B). Morphometric and statistical analysis clearly demonstrated that such size disparities were significantly larger than normal among 2-cell conceptuses (Figure 5C). About 28% of CP-treated 2-cell conceptuses underwent second cleavage when they were washed and transferred into fresh medium, but the majority became arrested either at 2-cell stage or during the second cleavage so as to yield 3-cell stages (Table 2). To exclude a possibility that the CP reduces energy production of the mitochondrion then affects development of the mouse conceptuses, living cell staining using an oxidation-sensitive, mitochondrion-specific fluorescent dye was carried out. Even after 24 hours treatment of CP, the treated 2-cell conceptus did not display any visual sign of the functional retardation of mitochondrion (Figure 5D and 5E). In longer term, impaired mitochondrial translation could eventually affect the respiratory function because all 13 mitochondrion-encoded proteins are indispensable to the electron transport chain and oxidative phosphorylation, the aberrant cleavage pattern of zygotes seems to be a direct result of failure of the mitochondrial translation in short term.

**Table 2:** Effect of chloramphenicol on the second cleavage of mouse conceptuses

stage	2-cell	3-cell	4∼8-cell	total
CP	65	26	36	127
vehicle	2	0	80	82

Late pronuclei stage zygotes (around E0.75) were cultured for 24 hours in presence of either 200 µg/mL of chloramphenicol (CP) or 1% of DMSO (vehicle) as vehicle control. The cleaved 2-cell conceptuses were then selected, washed and cultured in fresh medium for further 24 hours. Numbers of conceptuses reaching denoted cleavage stages were counted and presented. Number of independent trials: 15.

## Discussion

### The mtrRNAs are subcellularly distributed in the mouse oocyte

Contrary to the uniform and amorphous appearance of the mouse secondary oocyte except in the region of the MII spindle, certain gene products are localized within its cytoplasm [17], [18]. Whether these products are related to the cleavage pattern of the zygote is not clear. This study shows that rRNAs derived from mitochondria are also localized in the mouse oocyte. This is particularly interesting because mtrRNA is one of the major components of the germinal body in various metazoa including *Drosophila* [12], [55] and *Xenopus* [16]. The notion that the mammalian oocyte also has germ plasm or, indeed, exhibits any pre-patterning tends to be dismissed [19]–[21]. However, the occurrence of localized mtrRNAs in the mouse oocyte and zygote implies that developmental strategies in mammals may have aspects in common with other metazoa. While loss-of-function type experiments show that these mtrRNAs are crucial for segregation of germ cells in certain metazoa [56], the present study implicates them in establishing a proper first cleavage pattern in mice.

### The mtrRNAs are acting through translational contribution

CP-treatment of zygotes reveals that the mtrRNA is acting through its own translational contribution rather than newly uncovered functionality of RNA like microRNA or ribozyme. In *Drosophila*, translation of a gene named “*germ-cell less* (*gcl*)”, whose loss-of-function leads to failure of pole cell formation, is known to be dependent on mtrRNA [57]. This rather unusual translation of nuclear genome-encoded mRNA by mtrRNA also takes place in mammalian sperm during capacitation [58] raising the possibility that similar translational activity might be initiated upon fertilization of mouse oocytes. In *Drosophila*, maternally deposited mRNA of *gcl* is translated in association with posteriorly localized mtrRNA through which the cytoplasm of posterior pole becomes the germ plasm. In the case of mouse, inhibition of the mtrRNA's contribution to translation perturbs positioning of the first cleavage plane.

The size disparity between blastomeres of the 2-cell conceptus is not significant in non-treated or vehicle control culture unless morphometric analysis is performed, when a small minority shows visible size difference under a microscope. These unequally cleaved 2-cell conceptuses however develop normally till at least the blastocyst stage. In contrast, a high proportion of CP-treated 2-cell conceptuses arrested at 2- or 3-cell stage regardless of whether they showed obvious differences in blastomere size. The mechanism whereby the first cleavage plane is established in the mouse zygote is as yet unknown, but seems to tolerate fluctuation in its positioning. As the CH-treated zygote arrested during the first cleavage, most of protein components, which are essential for the mitosis, should be dependent on *de novo* global cytoplasmic translation. Mitochondrial translation, on the other hand, accounts for less than 2% of total translation of the mouse oocytes and zygotes [59], small number of unidentified protein factors, which are required for proper establishment of the cleavage plane, might rely on the mtrRNA to their translation. Some manipulation experiments on the mouse zygotes, such as removing either animal or vegetal hemisphere region, suggest that localized factors at animal hemisphere direct the mitotic spindle and therefore define the plane of first cleavage [60]. As the 16S rRNAs are predominantly distributed at the animal hemisphere of MII oocytes and zygotes, the mtrRNA could be one such factor. Hence, maternally deposited mRNAs and their mtrRNA-dependent translation might be involved in stabilizing the position of the first cleavage plane and the proper segregation of the cytoplasmic components of the zygote.

A series of studies shows that the mouse pronuclear zygotes are bilaterally symmetrical and this bilateral symmetry extends to the blastocyst [27]. This bilateral symmetry relates neither to the sperm entry point nor the pronuclei distribution, supporting the assumption that the morphological significance might be a reflection of intrinsic organization of the oocyte [61]. It has been claimed that the entry point of sperm determines the prospective first cleavage plane of mouse zygote [62], but this has been challenged by other findings arguing that the position of first cleavage plane has been specified intrinsically [31], [63] or epigenetically [64]–[66] rather than determined by an external cue. That inhibition of a maternal process such as mitochondrial translation leads to disruption of zygote cleavage, and haploid parthenotes show similar distribution pattern and dynamics of the mtrRNAs to the zygote further support the possibility of pre-patterning of the mouse oocytes.

A transmission electron microscopy showed that the CP treatment does not affect the amount of ribosomes in both cytoplasm and mitochondria of the mouse conceptuses [32]. Taken together with present result showing the CP treatment has no impact on the respiratory function of mitochondrion, the failure of mitochondrial translation should be responsible for the aberrant cleavage. Which genes are dependent on mtrRNA for translation in mouse oocytes and zygotes is an intriguing question that needs to be addressed.

### The mtrRNAs are extra-mitochondrial

ATP6 and Cox1 expression were not detectable from MII oocyte to 8-cell morula either because the ISH used here was not sensitive enough to detect their transcripts or the DIG-labeled probes were unable to penetrate into the mitochondria. A previous study using blot hybridization showed that the amount of mtrRNAs and Cox1 mRNA remains low until just before the second cleavage and increase at least 25 times thereafter by the early blastocyst stage [39]. The study also revealed that the actual number of molecules of Cox1 mRNA in 8-cell morula is more than one-fifth and one-tenth to that of mtrRNA in MII oocyte and 8-cell morula respectively, and the amount of mitochondrial mRNAs is about 10% of total amount of poly(A)+ RNAs at the 8-cell morula stage [39]. Assuming that there are such abundant transcripts in the 8-cell morula requiring plentiful energy to sustain growth, it is unlikely that the ISH is not sensitive enough to detect mRNAs of ATP6 and Cox1. In fact, an ISH by antisense probe to the mEMK, which is nuclear genome-encoded single copy gene, successfully revealed its expression dynamics around mouse fertilization, as previously reported using RT-PCR [46]. Whereas a mitochondrial Cytochrome *b* DIG-labeled antisense probe, which is known to hybridize well on mouse brain sections (Wang 2003, personal communication), failed to give staining on the MII oocyte and 8-cell morula by this ISH (data not shown). These results support the view that the ISH methodology used was able to detect RNAs in the cytoplasm but not inside the mitochondria of whole mount tissue.

Sense probes of 16S rRNA (Figure S2) and other mitochondrial genes (data not shown) gave either background level staining intensity (Figure S2A) or no gold colloidal particle deposition (Figure S2C), indicating that the rRNA antisense staining did not come from the hybridization between RNA probes and mitochondrial genomic DNAs, even if the probes penetrated into the mitochondria. Since the transcription of mitochondrial DNA is bidirectional, it also indicates that no detectable hybridization took place between the probes and pre-processed transcripts. In line with several other reports using mitochondrion-specific fluorescent dye [48], [49], anti-mtHSP70 immunofluorescence showed relatively even distribution of mitochondria throughout the cytoplasm of MII oocytes. These results contradict one particular observation employing a combination of living oocyte laser confocal microscopy and mitochondrion-specific fluorescent dye, in which an animal hemisphere-predominat polarized distribution of non-spindle mitochondria has been reported [67]. The author attributed the contradiction to a superior ability of the confocal microscopy over a conventional microscopy for viewing entire oocyte. However, it also might be possible that these non-spindle mitochondria have selective membrane permeability to the dye in a living condition, hence the strong fluorescent. Interestingly, the polarized distribution of mitochondria in the living oocyte is strikingly similar to that of 16S rRNAs reported here. As the distributions of mitochondria and 16S rRNAs do not coincide, a likely explanation for this discrepancy would be that either the ISH used here detects mtrRNAs in a subset of mitochondria, which increase membrane permeability and localize animal hemisphere, or the mtrRNAs are translocated from mitochondria, which has been found to be the case in *Drosophila* [12], [13], sea urchin [15] and *Xenopus* [16]. The notion of extra-mitochondrial mtrRNA in the mouse oocyte is further ensured by the combination of ISH and SEM. The method employed here for the ISH-SEM was a pre-embedding hybridization and thus, it would be fewer gold colloidal particles inside endoplasmic organelle if the DIG-labeled probes have difficulties to penetrate membrane of the organelle. That is the exact case in terms of the mitochondria of MII oocytes. Moreover, the ISH-SEM also reveals the existence of cytoplasmic mtrRNAs, which are distributed in certain area of the oocyte but not all over the oocyte.

In mice and humans, the 16S rRNAs are translocated into the sperm nucleus from mitochondria during spermatogenesis [68], establishing a precedent for their presence extra-mitochondrially in mammals in certain circumstances. In *Drosophila,* the extra-mitochondrial mtrRNAs are translocated onto the surface of the polar granule, and this translocation is regulated under the posterior group genes [55], especially under *oskar* (*osk*) [69]. The polar granule itself is composed of *osk* protein together with products of *vasa* (*vas*) and *tudor* (*tud*), other major members of the posterior group genes [70]. An exact mechanism underlining the polar granule formation is as yet unknown [4], a common assumption is that an active translocation and aggregation of the components including osk, vas and tud is completed during oogenesis then mtrRNAs are translocated and associated with the granules in early embryogenesis [12].

However another interesting possibility becomes apparent. Transmission electron microscopy of the mouse Graafian follicle revealed that cristae-containing electron-dense bodies, which morphologically resemble the germinal bodies of other metazoa, are derivatives of mitochondria [71]. These derivatives lack an outer membrane and are gradually condensing into electron-dense bodies. It is also suggested that the transformed mitochondria might be precursors or platforms of the germinal body-like structure [72]. In any case, degradation or lack of outer membrane seems reasonable explanation for how DIG-labeled probes easily access to the mtrRNAs. It also might explain the similarity of disribution pattern between the mitochondria in a living oocyte [67] and the mtrRNAs in this report. Elucidating whether the electron-dense bodies are actually attaching the mtrRNA on their surface as well as *bona fide* composition and function of these cytoplasmic mitochondrial translation machinery are obvious next steps to be taken.

## Supporting Information

Figure S1 Expression and distribution of mitochondrial RNAs and mEMK in the mouse oocytes and zygotes. Distribution of two mitochondrial rRNAs and expression of two mitochondrial genes along with nucleus-encoded mEMK are examined by ISH in the MII oocytes and various stages of zygotes. The ISH staining intensity of 12S rRNA (A) is weaker than that of 16S rRNA (B) in the MII oocyte and zygote. ATP6 (C) and Cox1 (D) expression are undetectable during these stages. To illustrate a differential expression level of 16S rRNA and Cox1, 8-cell stage conceptuses are shown on fourth column. Transcripts of mEMK (E) are ubiquitous in the MII oocyte and zygote then diminished after the first cleavage. Durations of colour reaction: 30 minutes for mtrRNAs, 2 hours for mitochondrial mRNAs and 1 hour for mEMK. Bar = 25 µm
(4.26 MB TIF)

Figure S2 16S rRNA sense probe ISH for negative control. Sense (A, C) and antisense (B, D) probes of 16S rRNA were applied to the MII oocytes and visualized via either alkaline phosphatase (AP) substrate BCIP/NBT (A, B) or gold colloidal particle conjugated anti-DIG antibody (C, D). For AP colour reaction, the hybridized and AP conjugated anti-DIG antibody applied samples were embedded in agarose then incubated in the substrate solution in a same well for 35 minutes. Bar = 10 µm For electron microscopy, the hybridized samples were processed as described in the Materials and Methods section. SEM (C, D left panel) and backscattered (C, D right panel) images from same fields are presented side-by-side. Gold colloidal particles are seen as white dots with shadows in a backscattered image (D right panel). Bar = 0.5 µm
(6.37 MB TIF)

Table S1 Mean size and size difference of paired 2-cell stage blastomeres. This table is the raw data set to Figure 5C. Late pronuclei stage zygotes (around E0.75) were cultured for 24 hours in presence of either 200 µg/mL of chloramphenicol (CP200) or 1% of DMSO as vehicle control (vehicle). The cleaved 2-cell conceptuses were selected, fixed and photographed. Outlines of each blastomere are traced on the microphotographs then converted to the areas using ImageJ software. Pair values of the area are processed to calculate “Mean size of paired blastomeres (Mean Size)” and “Size difference between paired blastomeres (Size Diff.)” and presented. Unit: µm^2^
(0.02 MB XLS)

## References

[1] Wodarz A (2002). Establishing cell polarity in development.. Nat Cell Biol.

[2] Schupbach T, Roth S (1994). Dorsoventral patterning in *Drosophila* oogenesis.. Curr Opin Genet Dev.

[3] Rushlow C, Arora K (1990). Dorsal ventral polarity and pattern formation in the *Drosophila* embryo.. Semin Cell Biol.

[4] Mahowald AP (2001). Assembly of the *Drosophila* germ plasm.. Int Rev Cytol.

[5] Ding D, Lipshitz HD (1993). Localized RNAs and their functions.. Bioessays.

[6] Bashirullah A, Cooperstock RL, Lipshitz HD (1998). RNA localization in development.. Annu Rev Biochem.

[7] King ML, Zhou Y, Bubunenko M (1999). Polarizing genetic information in the egg: RNA localization in the frog oocyte.. Bioessays.

[8] Mowry KL, Cote CA (1999). RNA sorting in *Xenopus* oocytes and embryos.. Faseb J.

[9] Kloc M, Bilinski S, Chan AP, Allen LH, Zearfoss NR (2001). RNA localization and germ cell determination in *Xenopus*.. Int Rev Cytol.

[10] Rand K, Yisraeli J (2001). RNA localization in *Xenopus* oocytes.. Results Probl Cell Differ.

[11] Okada M, Kleinman IA, Schneiderman HA (1974). Restoration of fertility in sterilized *Drosophila* eggs by transplantation of polar cytoplasm.. Dev Biol.

[12] Kobayashi S, Amikura R, Okada M (1993). Presence of mitochondrial large ribosomal RNA outside mitochondria in germ plasm of *Drosophila melanogaster*.. Science.

[13] Amikura R, Kashikawa M, Nakamura A, Kobayashi S (2001). Presence of mitochondria-type ribosomes outside mitochondria in germ plasm of *Drosophila* embryos.. Proc Natl Acad Sci U S A.

[14] Kobayashi S, Okada M (1989). Restoration of pole-cell-forming ability to u.v.-irradiated *Drosophila* embryos by injection of mitochondrial lrRNA.. Development.

[15] Ogawa M, Amikura R, Akasaka K, Kinoshita T, Kobayashi S (1999). Asymmetrical distribution of mitochondrial rRNA into small micromeres of sea urchin embryos.. Zoo Sci.

[16] Kobayashi S, Amikura R, Mukai M (1998). Localization of mitochondrial large ribosomal RNA in germ plasm of *Xenopus* embryos.. Curr Biol.

[17] Vinot S, Le T, Maro B, Louvet-Vallée S (2004). Two PAR6 proteins become asymmetrically localized during establishment of polarity in mouse oocytes.. Curr Biol.

[18] Antczak M, Van Blerkom J (1997). Oocyte influences on early development: the regulatory proteins leptin and STAT3 are polarized in mouse and human oocytes and differentially distributed within the cells of the preimplantation stage embryo.. Mol Hum Reprod.

[19] Hiiragi T, Louvet-Vallée S, Solter D, Maro B (2006). Embryology: does prepatterning occur in the mouse egg?. Nature.

[20] Hiiragi T, Alarcon VB, Fujimori T, Louvet-Vallée S, Maleszewski M (2006). Where do we stand now? mouse early embryo patterning meeting in Freiburg, Germany (2005).. Int J Dev Biol.

[21] Hiiragi T, Solter D (2006). Fatal flaws in the case for prepatterning in the mouse egg.. Reprod Biomed Online.

[22] Zernicka-Goetz M (1998). Fertile offspring derived from mammalian eggs lacking either animal or vegetal poles.. Development.

[23] Mulnard JG, Puissant F (1984). Development of mouse embryos after ultracentrifugation at the pronuclei stage.. Arch Biol.

[24] Evsikov SV, Morozova LM, Solomko AP (1994). Role of ooplasmic segregation in mammalian development.. Roux's Arch Dev Biol.

[25] Gardner RL (1999). Scrambled or bisected mouse eggs and the basis of patterning in mammals.. Bioessays.

[26] Gardner RL (2006). Weaknesses in the case against prepatterning in the mouse.. Reprod Biomed Online.

[27] Gardner RL, Davies TJ (2006). An investigation of the origin and significance of bilateral symmetry of the pronuclear zygote in the mouse.. Hum Reprod.

[28] Zernicka-Goetz M (2005). Developmental cell biology: cleavage pattern and emerging asymmetry of the mouse embryo.. Nat Rev Mol Cell Biol.

[29] Zernicka-Goetz M (2006). The first cell-fate decisions in the mouse embryo: destiny is a matter of both chance and choice.. Curr Opin Genet Dev.

[30] Matova N, Cooley L (2001). Comparative aspects of animal oogenesis.. Dev Biol.

[31] Davies TJ, Gardner RL (2002). The plane of first cleavage is not related to the distribution of sperm components in the mouse.. Hum Reprod.

[32] Pikó L, Chase DG (1973). Role of the mitochondrial genome during early development in mice. Effects of ethidium bromide and chloramphenicol.. J Cell Biol.

[33] Aoki F, Hara KT, Schultz RM (2003). Acquisition of transcriptional competence in the 1-cell mouse embryo: requirement for recruitment of maternal mRNAs.. Mol Reprod Dev.

[34] Latham KE, Solter D, Schultz RM (1992). Acquisition of a transcriptionally permissive state during the 1-cell stage of mouse embryogenesis.. Dev Biol.

[35] Poot M, Zhang YZ, Kramer JA, Wells KS, Jones LJ (1996). Analysis of mitochondrial morphology and function with novel fixable fluorescent stains.. J Histochem Cytochem.

[36] Gardner RL (2002). Experimental analysis of second cleavage in the mouse.. Hum Reprod.

[37] Abramoff MD, Magelhaes PJ, Ram SJ (2004). Image processing with ImageJ.. Biophotonics Int.

[38] Rasband WS (1997). ImageJ. U S National Institutes of Health, Bethesda, Maryland, USA.. http://rsb.info.nih.gov/ij/.

[39] Pikó L, Taylor KD (1987). Amounts of mitochondrial DNA and abundance of some mitochondrial gene transcripts in early mouse embryos.. Dev Biol.

[40] Ko MS, Kitchen JR, Wang X, Threat TA, Hasegawa A (2000). Large-scale cDNA analysis reveals phased gene expression patterns during preimplantation mouse development.. Development.

[41] Ninomiya Y, Davies TJ, Gardner RL (2005). Experimental analysis of the transdifferentiation of visceral to parietal endoderm in the mouse.. Dev Dyn.

[42] Tokuyasu KT (1986). Application of cryoultramicrotomy to immunocytochemistry.. J Microsc.

[43] Ichinose S, Yamagata K, Sekiya I, Muneta T, Tagami M (2005). Detailed examination of cartilage formation and endochondral ossification using human mesenchymal stem cells.. Clin Exp Pharmacol Physiol.

[44] Drewes G, Ebneth A, Preuss U, Mandelkow EM, Mandelkow E (1997). MARK, a novel family of protein kinases that phosphorylate microtubule-associated proteins and trigger microtubule disruption.. Cell.

[45] Inglis JD, Lee M, Hill RE (1993). Emk, a protein kinase with homologs in yeast maps to mouse chromosome 19.. Mamm Genome.

[46] Vinot S, Le T, Ohno S, Pawson T, Maro B (2005). Asymmetric distribution of PAR proteins in the mouse embryo begins at the 8-cell stage during compaction.. Dev Biol.

[47] Green JM, Gu L, Ifkovits C, Kaumaya PT, Conrad S (1995). Generation and characterization of monoclonal antibodies specific for members of the mammalian 70-kDa heat shock protein family.. Hybridoma.

[48] Tokura T, Noda Y, Goto Y, Mori T (1993). Sequential observation of mitochondrial distribution in mouse oocytes and embryos.. J Assist Reprod Genet.

[49] Van Blerkom J, Runner MN (1984). Mitochondrial reorganization during resumption of arrested meiosis in the mouse oocyte.. Am J Anat.

[50] Perkins GA, Frey TG (2000). Recent structural insight into mitochondria gained by microscopy.. Micron.

[51] Frey TG, Mannella CA (2000). The internal structure of mitochondria.. Trends Biochem Sci.

[52] Flach G, Johnson MH, Braude PR, Taylor RA, Bolton VN (1982). The transition from maternal to embryonic control in the 2-cell mouse embryo.. Embo J.

[53] Van Blerkom J (1981). Structural relationship and posttranslational modification of stage-specific proteins synthesized during early preimplantation development in the mouse.. Proc Natl Acad Sci U S A.

[54] Johnson MH (1981). The molecular and cellular basis of preimplantation mouse development.. Biol Rev Camb Philos Soc.

[55] Ding D, Whittaker KL, Lipshitz HD (1994). Mitochondrially encoded 16S large ribosomal RNA is concentrated in the posterior polar plasm of early *Drosophila* embryos but is not required for pole cell formation.. Dev Biol.

[56] Iida T, Kobayashi S (1998). Essential role of mitochondrially encoded large rRNA for germ-line formation in *Drosophila* embryos.. Proc Natl Acad Sci U S A.

[57] Amikura R, Sato K, Kobayashi S (2005). Role of mitochondrial ribosome-dependent translation in germline formation in *Drosophila* embryos.. Mech Dev.

[58] Gur Y, Breitbart H (2006). Mammalian sperm translate nuclear-encoded proteins by mitochondrial-type ribosomes.. Genes Dev.

[59] Cascio SM, Wassarman PM (1981). Program of early development in the mammal: synthesis of mitochondrial proteins during oogenesis and early embryogenesis in the mouse.. Dev Biol.

[60] Plusa B, Grabarek JB, Piotrowska K, Glover DM, Zernicka-Goetz M (2002). Site of the previous meiotic division defines cleavage orientation in the mouse embryo.. Nat Cell Biol.

[61] Gardner RL, Davies TJ (2003). The basis and significance of pre-patterning in mammals.. Philos Trans R Soc Lond B Biol Sci.

[62] Piotrowska K, Zernicka-Goetz M (2001). Role for sperm in spatial patterning of the early mouse embryo.. Nature.

[63] Gardner RL, Davies TJ (2003). Is the plane of first cleavage related to the point of sperm entry in the mouse?. Reprod Biomed Online.

[64] Motosugi N, Bauer T, Polanski Z, Solter D, Hiiragi T (2005). Polarity of the mouse embryo is established at blastocyst and is not prepatterned.. Genes Dev.

[65] Motosugi N, Dietrich JE, Polanski Z, Solter D, Hiiragi T (2006). Space asymmetry directs preferential sperm entry in the absence of polarity in the mouse oocyte.. PLoS Biol.

[66] Hiiragi T, Solter D (2004). First cleavage plane of the mouse egg is not predetermined but defined by the topology of the two apposing pronuclei.. Nature.

[67] Calarco PG (1995). Polarization of mitochondria in the unfertilized mouse oocyte.. Dev Genet.

[68] Villegas J, Araya P, Bustos-Obregon E, Burzio LO (2002). Localization of the 16S mitochondrial rRNA in the nucleus of mammalian spermatogenic cells.. Mol Hum Reprod.

[69] Kobayashi S, Amikura R, Nakamura A, Saito H, Okada M (1995). Mislocalization of *oskar* product in the anterior pole results in ectopic localization of mitochondrial large ribosomal RNA in *Drosophila* embryos.. Dev Biol.

[70] Hay B, Ackerman L, Barbel S, Jan LY, Jan YN (1988). Identification of a component of *Drosophila* polar granules.. Development.

[71] Reunov A (2004). Is there a germ plasm in mouse oocytes?. Zygote.

[72] Reunov A (2006). Structures related to the germ plasm in mouse.. Zygote.

