# Supplementary figures and images for: Subcellular Distribution of Mitochondrial Ribosomal RNA in the Mouse Oocyte and Zygote
Source: PLoS One. 2007 Nov 28;2(11):e1241. doi: 10.1371/journal.pone.0001241 (PMC2082410; doi:10.1371/journal.pone.0001241)

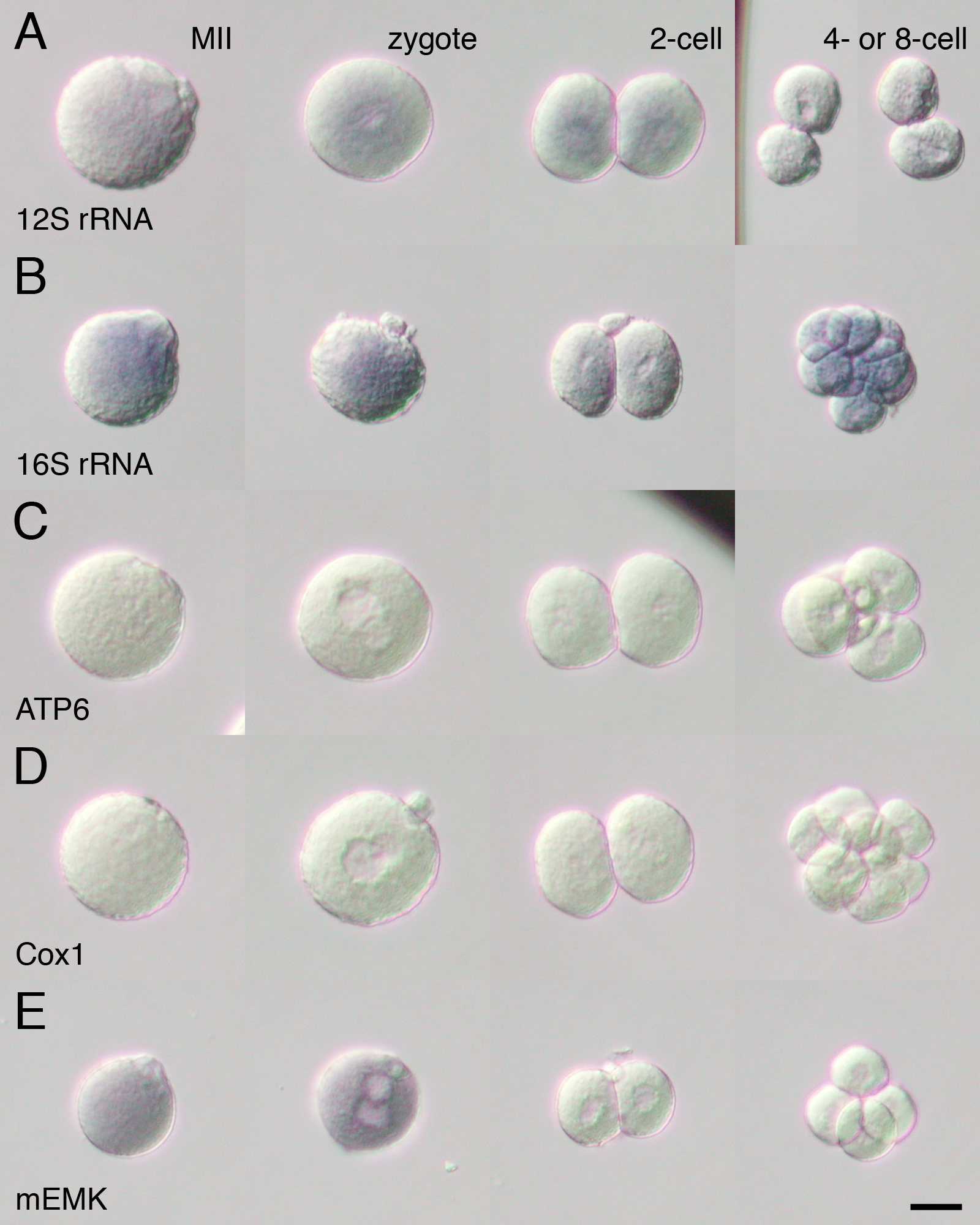

Supplement: Figure S1 — Expression and distribution of mitochondrial RNAs and mEMK in the mouse oocytes and zygotes. Distribution of two mitochondrial rRNAs and expression of two mitochondrial genes along with nucleus-encoded mEMK are examined by ISH in the MII oocytes and various stages of zygotes. The ISH staining intensity of 12S rRNA (A) is weaker than that of 16S rRNA (B) in the MII oocyte and zygote. ATP6 (C) and Cox1 (D) expression are undetectable during these stages. To illustrate a differential expression level of 16S rRNA and Cox1, 8-cell stage conceptuses are shown on fourth column. Transcripts of mEMK (E) are ubiquitous in the MII oocyte and zygote then diminished after the first cleavage. Durations of colour reaction: 30 minutes for mtrRNAs, 2 hours for mitochondrial mRNAs and 1 hour for mEMK. Bar = 25 µm (4.26 MB TIF) [file pone.0001241.s001.tif]

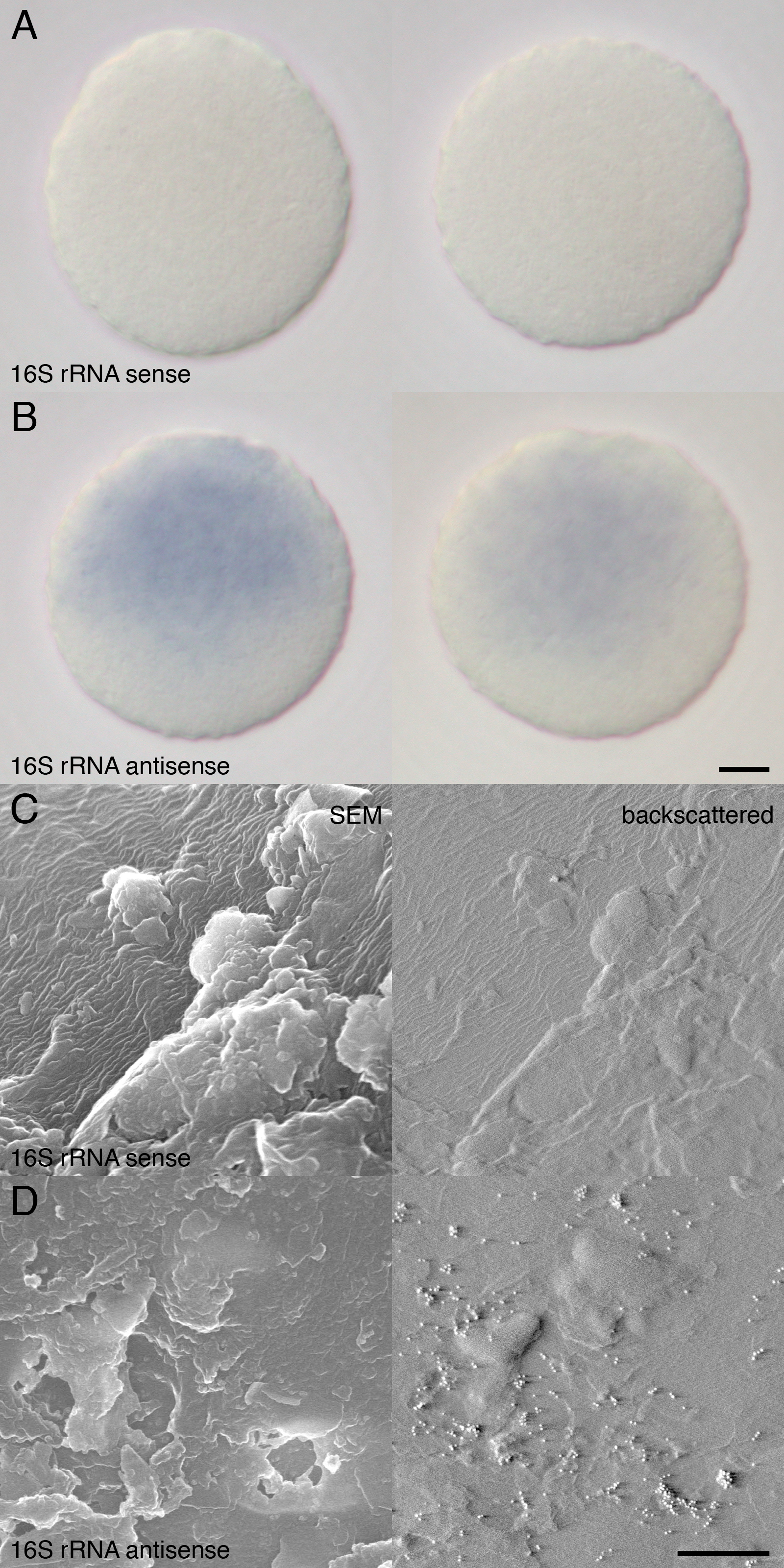

Supplement: Figure S2 — 16S rRNA sense probe ISH for negative control. Sense (A, C) and antisense (B, D) probes of 16S rRNA were applied to the MII oocytes and visualized via either alkaline phosphatase (AP) substrate BCIP/NBT (A, B) or gold colloidal particle conjugated anti-DIG antibody (C, D). For AP colour reaction, the hybridized and AP conjugated anti-DIG antibody applied samples were embedded in agarose then incubated in the substrate solution in a same well for 35 minutes. Bar = 10 µm For electron microscopy, the hybridized samples were processed as described in the Materials and Methods section. SEM (C, D left panel) and backscattered (C, D right panel) images from same fields are presented side-by-side. Gold colloidal particles are seen as white dots with shadows in a backscattered image (D right panel). Bar = 0.5 µm (6.37 MB TIF) [file pone.0001241.s002.tif]
